# Percentage oleic acid is inversely related to percentage arachidonic acid in total lipids of rat serum

**DOI:** 10.1186/1476-511X-12-40

**Published:** 2013-03-25

**Authors:** Arne Torbjørn Høstmark, Anna Haug

**Affiliations:** 1 University of Oslo, Norway, Institute of Health and Society, Section of Preventive Medicine and Epidemiology, Box 1130, Blindern 0318, Oslo, Norway; 2 The Norwegian University of Life Sciences, Department of Animal and Aquacultural Sciences, Box 5003, 1432 Ås, Norway

## Abstract

**Background:**

Since many health effects of oils rich in oleic acid (18:1, n-9) seem to be opposite those of arachidonic acid (20:4, n-6), i.e. concerning cardiovascular risk, we examined whether % 18:1 might be negatively associated with % 20:4.

**Methods:**

Fatty acid separation by gas chromatography was performed in total serum lipids of 36 male rats. Using bivariate correlations and multiple linear regressions we studied the association between oleic acid and arachidonic acid.

**Results:**

We found an inverse relationship (r = -0.885, p < 0.001; n = 36) between percentages of 18:1 and 20:4 in total lipids of rat serum, persisting when controlling for the other fatty acids measured. In a multiple linear regression model with % 20:4 as the dependent variable and percentages of the other fatty acids entered simultaneously as independents, oleic acid and linoleic acid contributed most to predict % 20:4. Per cent 20:4 correlated negatively (p< 0.01) with a Delta-9 desaturase index, i.e. the (18:1)/(18:0) ratio, and with the (20:4)/(18:2) ratio, estimating desaturases/elongase.

**Conclusions:**

Percentages of 18:1 and 20:4 seem to be inversely related and desaturase/elongase inhibition could be involved. The results might partly explain positive health effects of foods rich in oleic acid.

## Background

It is widely accepted that oleic acid (18:1, n-9), and oleic acid rich foods such as olive oil may have many beneficial health effects. Among such effects are improved insulin sensitivity, and endothelium-dependent flow-mediated vasodilatation [1], lowering of LDL cholesterol [2,3] and an increase in HDL cholesterol [4]. If LDL lipids are enriched in oleic acid, the particles will be less liable to be oxidized [5], a property that is of significance for the normal metabolism of LDL [6]. Furthermore, intake of oleic acid seems to be associated with reduced blood pressure [7]. Thus, many of the effects of oleic acid may serve to reduce the risk of cardiovascular diseases. Additionally, the fatty acid may have anticarcinogenic and anti inflammatory effects [8-10].

Although beneficial effects of oils rich in oleic acid have been reported, the mechanisms by which such oils might have beneficial health effects are still incompletely understood. Various antioxidants present in e.g. virgin olive oil, as well as the high content of oleic acid, could partly explain the health effects.

When considering the reported beneficial health effects of oils rich in oleic acid, it occurred to us that many of the positive effects would be anticipated if the fatty acid works to counteract effects of arachidonic acid (20:4, n-6). This fatty acid is formed in the body from linoleic acid (18:2, n-6), a major constituent in many plant oils, and is converted by cyclooxygenase and lipoxygenase into various eicosanoids, i.e. prostacyclines, thromboxanes and leukotrienes [11]. Arachidonic acid derived thromboxane A_2_ (TXA_2_) and leukotriene B_4_ have strong proinflammatory and prothrombotic properties [12,13]. Furthermore, endocannabinoides, which are derived from arachidonic acid, may have a role in adiposity [14].

An interaction between oleic acid and arachidonic acid was suggested several years ago in the rat [15]. More recently, Cicero et al. [5] showed in human subjects that supplementation with a high dose of olive oil for 3 weeks resulted in an increase in LDL oleic acid and a decrease in linoleic and arachidonic acid. Also in chicken breast muscle a negative 18:1 vs. 20:4 association was observed [16].

One mechanism by which oleic acid could counteract those of arachidonic acid is to reduce the relative abundance of arachidonic acid in serum and tissues. Conceivably, increased supply of 18:1 might reduce that of 20:4 by pure mass action. This suggestion raises the question of whether an exchange of 20:4 for 18:1 is specific for this couple of fatty acids.

Inverse regulation could also be effected through more specific metabolic feedback regulation. For example, a reduced percentage of arachidonic acid would be expected if oleic acid inhibits Delta-6 desaturase, Elongase-5 (Elovl-5) and/or Delta-5 desaturase, the enzymes governing formation of arachidonic acid from linoleic acid. Conversely, inhibition by arachidonic acid of Delta-9 desaturase should lower percentage oleic acid, and previous studies suggest that this latter mechanism might take place [17].

It seems that the Delta-9 desaturases are of considerable physiological significance. Thus, regulation of the amount of mono unsaturated fatty acids (MUFA) has the potential to affect a variety of key physiological variables, such as insulin sensitivity, metabolic rate, adiposity, atherosclerosis, cancer and obesity [17,18].

The aim of this work was to examine whether there exists an inverse relationship between oleic acid and arachidonic acid, as studied in total lipids of rat serum. Since there are numerous intercorrelations between percentages of various types of fatty acids, we examined whether the bivariate association prevailed when controlling for other fatty acids. In an attempt to elucidate whether desaturase inhibition is involved, we examined if there might be an inverse relationship between oleic acid and a crude Delta-5/6 desaturase index, i.e. the (20:4)/(18:2) ratio, and between arachidonic acid and a Delta-9 desaturase index, i.e. the (18:1)/(18:0) ratio.

## Results

### Bivariate association between percentage oleic acid and arachidonic acid in total serum lipids

**Figure 1:**
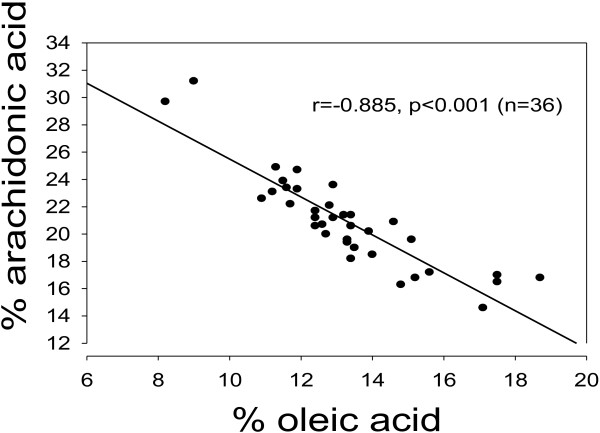
**Association between percentages of oleic acid (18:1, n-9) and arachidonic acid (20:4, n-6) in total lipids of rat serum. **p < 0.001 for the negative association. Note broken axes.

There was an inverse relationship (r = -0.885, p < 0.001; n = 36) between percentages of 18:1 and 20:4 in total lipids of rat serum (Figure 1).

### Linear regression models to evaluate the relationship between oleic acid and arachidonic acid in serum lipids, as influenced by other fatty acids

**Figure 2:**
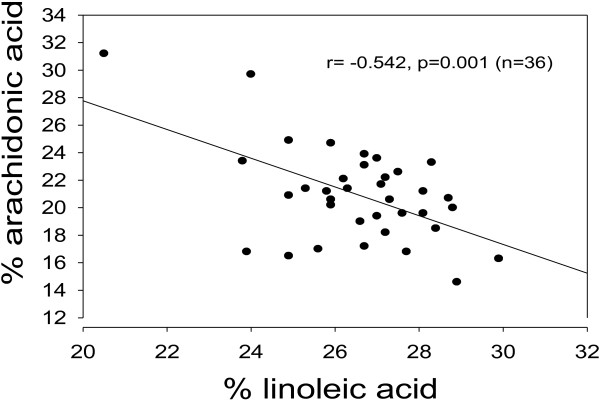
**Association between percentages of linoleic acid (18:2, n-6) and arachidonic acid (20:4, n-6) in total lipids of rat serum. **p = 0.001 for the negative association. Note broken axes.

Both the absolute and relative amounts of many fatty acids are intercorrelated, and the inverse relationship between oleic acid and arachidonic acid could possibly be attributed to co variation with other fatty acids. Conceivably, increase in the percentage of one particular fatty acid must be accompanied by a reduction in the percentage of one or more of the other fatty acids. We accordingly studied the association between % 18:1 (main independent variable) and % 20:4 (dependent variable), using multiple linear regressions and controlling for percentages of the other fatty acids measured. As shown in Table 1, the successive addition of one more fatty acid to the list of independents, starting from myristic acid, had only a minor influence on the standardized regression coefficient (beta), and the inverse association between % oleic acid and % arachidonic acid prevailed in all regression models. When simultaneously entering percentages of all of the fatty acids determined it turned out that oleic acid and linoleic acid provided the greatest contribution to predict % 20:4, as judged from the beta values (results not shown). In accordance with the precursor/product relationship between linoleic acid and arachidonic acid, their relative amounts correlated negatively (Figure 2).

**Table 1:** Association between relative amount of arachidonic acid (dependent variable) and oleic acid (independent variable under investigation), as influenced by other fatty acids, linear regression

**Model**		**B (SE)**	**Beta**	**t**	**p**
1	No adjustment	-1.39(0.13)	-0.89	-11.1	<0.001
2	Model 4 + % 18:0	-1.33 (0.14)	-0.85	-9.6	<0.001
3	Model 2 + % 16:0	-1.17 (0,15)	-0.75	-8.0	<0.001
4	Model 3 + % 16:1	-1.33 (0.16)	-0.85	-8.2	<0.001
5	Model 4 + % 18:1	-1.06 (0.19)	-0.67	-5.6	<0.001
6	Model 5 + % 18:2	-0.93 (0.04)	-0.59	-25.0	<0.001
7	Model 6 + % 18:3	-0.91 (0.04)	-0.58	-24.9	<0.001
8	Model 7 + % 20:1	-0.92 (0.04)	-0.58	-24.7	<0.001
9	Model 8 + % 20:2	-0.89 (0.04)	-0.57	-24.5	<0.001
10	Model 9 + % 20:5	-0.89 (0.04)	-0.56	-23.9	<0.001
11	Model 10 + % 22:6	-0.98 (0.02)	-0.63	-49.0	<0.001

Dependent variable = % 20:4, n-6. B = unstandardized regression coefficient, Beta = standardized coefficient. Model 1 is without adjustment; Model 2 with adjustment for % 14:0; Model 3 = Model 2 + % 16:0; Model 4 = Model 3 + % 16:1; Model 5 = Model 4 + % 18:0; Model 6 = Model 5 + % 18:2; Model 7 = Model 6 + % 18:3; Model 8 = Model 7 + % 20:1; Model 9 = Model 8 + % 20:2; Model 10 = Model 9 + % 20:5; Model 11 = Model 10 + % 22:6.

### Is the inverse relationship between percentages of 18:1 and 20:4 related to feedback inhibition of desaturases?

**Figure 3:**
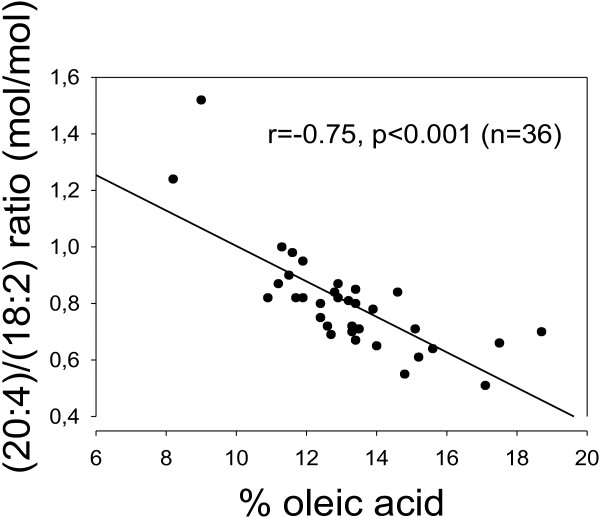
**A Delta-5 desaturase index as related to percentages of oleic acid in total lipids of rat serum. **% 18:1 (abscissa) was negatively correlated with a Delta-5 desaturase index, i.e. the (20:4)/(18:2) ratio (r = -0.75, p < 0.001). Note broken axes.

**Figure 4:**
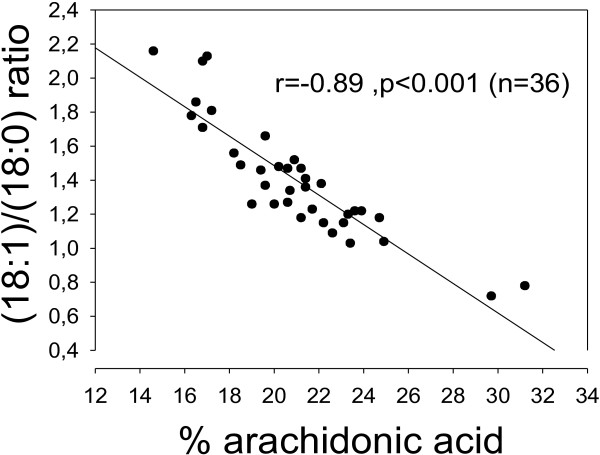
**A Delta-9 desaturase index as related to percentages of arachidonic acid in total lipids of rat serum. **% 20:4 was negatively correlated with a Delta-9 desaturase index, i.e. the (18:1)/(18:0) ratio (r = -0.89, p < 0.001). Note broken axes.

One explanation of the inverse oleic acid vs. arachidonic acid association could be that Delta-5 or 6 desaturases and/or Elongase-5 are inhibited by oleic acid, and/or conversely, that Delta-9 desaturase is inhibited by arachidonic acid. Using product/precursor ratios as crude estimates of desaturase activities, i.e. the (18:1)/(18:0) ratio for Delta-9 desaturase, and the (20:4)/(18:2) ratio to estimate Delta-5/6 desaturase and Elongase-5, we did find inverse relationships (r = -0.89, p < 0.001 for % 20:4 vs. the (18:1)/(18:0) ratio; and r = -0.75, p < 0.001 for % 18:1 vs. the (20:4)/(18:2) ratio; Figures 3 and 4).

## Discussion

The present results show that the relative abundance of arachidonic acid is inversely related to that of oleic acid, as observed in total lipids of rat serum. Surprisingly, reports focusing upon this particular relationship and possible health implications seem scarce. However, an interaction in the rat between oleic acid and linoleic acid, the precursor of arachidonic acid, was reported several years ago [17]. More recently, in a multi-center randomized cross-over study involving 200 healthy European subjects, Cicero et al. [5] showed that a 3 weeks supplementation with olive oil resulted in an increase in LDL oleic acid and a decrease in linoleic and arachidonic acid. The increase in oleic acid/linoleic acid ratio was accompanied by reduced levels of isoprostanes, biomarkers of oxidative stress. The finding of a negative correlation between oleic acid and arachidonic also in chicken breast muscle [16] could suggest that the inverse relationship between this couple of fatty acids exists in many species. Furthermore, in accordance with this suggestion, Kudo et al. [19] observed in mice treated with perfluorinated fatty acids, zenobiotics used as surfactants in various industrial products, that 18:1 was inversely related to 20:4 in hepatic phospholipids and total lipids.

The present study does not provide information to fully explain how the inverse association is brought about. One possibility is that the associations might have been caused by changes in the relative amount of other fatty acids. Conceivably, increase in the percentage of one particular fatty acid must be accompanied by a reduction in the percentage of one or more of the other fatty acids.

However, the inverse association between 18:1 and 20:4 prevailed with high significance in linear regression models when controlling for all of the other fatty acids measured. Furthermore, there were no major changes in the standardized regression coefficient for the inverse relationship when including an increasing number of the other fatty acids. It would appear, therefore, that the inverse association between these fatty acids is not caused by co-variation with other of the measured fatty acids. In theory, we cannot rule out the possibility that the association was due to co-variation with fatty acids which were below the threshold to be detected in our system, but we have no hypothesis to support this suggestion.

Since the aim of the present work was to study the possibility of an interaction between 18:1 and 20:4, we used relative amounts of the fatty acids rather than absolute values. The bivariate association between absolute values of oleic and arachidonic acids could be greatly different from those found when studying associations between their relative abundance. Conceivably, rats with increasing total amount of serum lipids could qualitatively have increasing amounts of all types of fatty acids, in spite of variation in how much each fatty acid was increased. However, as expected, the outcome presented in this work was confirmed when using multiple linear regression models, with absolute amount (mg/ml) of 20:4 as the dependent variable and that of 18:1 as the main independent, provided the analyses were controlled for the sum (mg/ml) of all fatty acids measured.

That 18:1 seems to have a special strong inverse association with 20:4 was suggested in the simultaneous multiple linear regression models used. In this analysis oleic acid and linoleic acid provided the greatest contribution to predict percentage arachidonic acid in total serum lipids, as judged from magnitudes of the standardized regression coefficients. The finding that percentage arachidonic acid also correlated negatively with that of linoleic is in accordance with the precursor-product relationship existing between these couple of fatty acids.

The finding of an inverse oleic acid vs. arachidonic acid association in our rats, which were fed pellets with a low content of oleic acid, raises the question of how the inverse association between percentages of the two fatty acids is governed. The present study does not clarify metabolic details to explain the inverse relationship between the relative abundance of two fatty acids. By pure mass action, increased supply of oleic acid might replace arachidonic acid in various compartments, such as in the lipids of cell membranes. This type of mechanism was previously suggested by the results of a diet trial in chicken [16]. We do not know, however, whether oleic acid by pure mass action has a particular ability to be exchanged for arachidonic acid, or whether this mechanism goes for other fatty acids as well.

Additionally, oleic acid seems to be a weak competitive inhibitor of cyclooxygenases [20], which catalyzes conversion of the C20 PUFAs arachidonic acid and eicosapentaenoic acid into prostaglandins, thromboxanes and leucotrienes [11].

Another mechanism serving to explain the inverse relationship could be that oleic acid acts as an inhibitor of Delta-5/6 desaturases and/or Elongase-5 (Elovl-5) so as to reduce the formation of arachidonic acid. Additionally, the possibility exists that arachidonic acid might inhibit the formation of oleic acid by inhibition of Delta-9 desaturase. Our results seem to be in accordance with the view that both of these mechanisms might take place since there was an inverse relationship between oleic acid and the Delta-5/6 desaturase-elongase index, and between arachidonic acid and the Delta-9 desaturase index. Possibly, inhibition by arachidonic acid of Delta-9 desaturase gene transcription might be involved, since previous studies suggest that PUFAs of both the n-6 and n-3 families can inhibit this transcription [18]. In the present study there was however no indication of a general inhibition by PUFAs of Delta-9 desaturase. To our knowledge, there are no previous reports suggesting that Delta-5 desaturase, Delta-6 desaturase or Elongase-5 (Elovl-5) may be inhibited by oleic acid. However, based upon results of a cross sectional human study, and cell culture experiments, Høstmark and Lunde [21] suggested that cheese might contain factors with desaturase inhibiting properties. From the present results it would appear that one of the cheese factors could be oleic acid. We emphasize, however, that the desaturase indexes used in this study are only crude estimates of the enzyme activities, and more direct methods are needed to confirm whether the suggested hypotheses are valid. The present results would seem to be in accordance with a previous report showing an increase in LDL oleic acid, and a decrease in LDL arachidonic acid, in response to ingesting 25 ml olive oil per day for 3 weeks [5].

Conceivably, also regulation of fatty acid uptake in the intestines, liver and other tissues, e.g. by various fatty acid transporters could be involved, as well as regulation of the catabolism of the fatty acids. Our study was not designed however to elucidate these questions. Finally, it is tempting to speculate whether genetic factors govern the inverse association between oleic acid and arachidonic acid observed among supposedly similar rats.

### Do the findings have health implications?

It would appear that many of the alleged positive health effects of oleic acid should be expected if oleic acid acts to counteract effects of, or reduce the relative amounts of, arachidonic acid. This latter fatty acid promotes inflammation and thrombosis, and thereby increases the risk of cardiovascular diseases [13]. Thromboembolic risk reduction by increasing the oleic acid intake might take place via a reduction in the percentage of arachidonic acid in phospholipids in platelet, thereby reducing the production of TXA_2_ and platelet aggregation. It has been reported that platelet aggregation and TXA_2_ release are decreased after exposure to olive oil, and this effect is accompanied by an increase in platelet membrane content of oleic acid and a reduction in arachidonic acid [6]. In addition, replacing LDL arachidonic acid by oleic acid should reduce the formation of oxidized LDL, since oleic acid is the least oxidizable of the unsaturated fatty acids [6]. We suggest that the consistent inverse relationship between percentages of oleic acid and arachidonic acid, observed when controlling for other fatty acids, could imply that increased percentage of oleic acid decreases that of arachidonic acid in various body compartments. The finding of an inverse relationship under various dietary conditions, and the present observed associations with desaturase indexes, could suggest that the relationship is governed by metabolic feedback regulation. It would appear that the relationship between the abundance of the two fatty acids can be appreciably altered by diet. Hypothetically, an increased supply of oleic acid might lower the percentage of arachidonic acid, and possibly reduce the risk of arachidonic acid associated conditions and diseases, such as inflammation and cardiovascular diseases. However, the present data are not sufficient to substantiate this hypothesis, and further studies are required to examine the possibility.

Whatever the mechanisms might be, the present results show a consistent inverse relationship between percentages of oleic acid and arachidonic acid. Further studies are required to appreciate the relevance of the inverse relationship, and to what extent there is a metabolic feedback regulation between the two fatty acids. Such studies are currently in progress, and our preliminary results in other species seem to confirm the general nature of the results obtained in this work.

## Conclusions

We have observed a consistent inverse relationship between the relative abundance of oleic acid and arachidonic acid in rat serum, prevailing after controlling for other fatty acids. The finding raises the question of whether a similar relationship exists in other species, and whether there might be a metabolic feedback regulation between the two fatty acids. Further studies are required to elucidate to what extent this inverse relationship might serve to explain health effects of foods rich in oleic acid.

## Methods

The rat trial was performed with the approval of the Regional Norwegian Ethics Committee and followed internationally recognized guidelines. Thus, the experiment was conducted in accordance with the laws and regulations controlling experiments/ procedures in live animals in Norway, i.e. the Animal Welfare Act of December 20. 1974 No 73, chapter VI, sections 20–22, and the Regulation on Animal Experimentation of January 15th 1996. Norway has signed and ratified The European Convention for the protection of Vertebrate Animals used for Experimental and other Scientific Purposes of March 18th 1986. The Norwegian legislation conforms in all respects with the basic requirements of this Convention and guidelines prepared in pursuance of it.

Male rats (Mol:Wist, L1, Skensved, Denmark) were kept in-house for a one week acclimatisation and fed ad libitum regular rat pellets RM1 from Special Diets Services, England (2.7% crude fat, 14.4% crude protein, 4.7% crude fibre and 6.0% crude ash). Distribution of fatty acids in the pellet diet (g/100 g pellets) was: lauric acid 0.02; myristic acid 0.14; palmitic acid 0.31; stearic acid 0.04; myristoleic acid 0.2; palmitoleic acid 0.09; oleic acid 0.77; linoleic acid 0.69; linolenic acid 0.06; arachidonic acid 0.13. A group of 36 rats (body weight 172 ± 1 g; mean ± SEM) was used to study the relationship between percentages of oleic acid and arachidonic acid in serum total lipids. The rats were given distilled water ad libitum.

### Blood and tissue sampling

In all groups, and after a 4–6 hour fast, venous blood was collected from the right dorso-lateral tail vein, using heparin-moistened syringes. Blood samples were centrifuged at 1750 × g for 10 min. and the supernatant was collected and frozen at -70°C.

The fatty acid profile was determined in serum total lipids. The lipids were extracted using n-butanol. Diheptadecanoyl-glycerophospho ethanolamine and butylated hydroxytoluene (Sigma Chemical, United Kingdom) were added as internal standard and antioxidant, respectively. Fatty acid methyl esters were quantified as mg fatty acid/g tissue, using gas liquid chromatography on a SP2330 column (Supelco Inc., Bellefonte, PA). A normal human serum sample was included to assess analytical performance. The results of the measurements are presented as weight percentage of total fatty acids. The following fatty acids were analyzed: Myristic acid (14:0), palmitic acid (16:0), palmitoleic acid (16:1, n-7), stearic acid (18:0), oleic acid (18:1, n-9), linoleic acid (18:2, n-6), linolenic acid (18:3, n-3), eicosenoic acid (20:1, n-9), eicosadienoic acid (20:2, n-6), arachidonic acid (20:4, n-6), eicosapentaenoic acid (20:5, n-3), and docosahexaenoic acid (22:6, n-3). The day to day coefficient of variation (n = 28) for 18:0; 18:1, n-9; 18:2, n-6; 20:4, n-6; 20:5, n-3; and 22:6, n-3 was 5.2, 6.2, 6.6, 9.6, 10.0, and 11.6%, respectively.

### Estimates of fatty acid desaturases

To estimate Delta-9 desaturase, we used the (18:1, n-9)/ (18:0) ratio. In the present article we may use the term “Delta-5 desaturase” for the (20:4, n-6)/(18:2, n-6) ratio, keeping in mind that this ratio also includes activities of Elongase-5 and Delta-6 desaturase.

### Statistical analysis

The relationship between percentages of fatty acids was assessed by correlation (Pearson) and linear regression, with % arachidonic acid as the dependent variable and % oleic acid as the independent variable under investigation. We made several regression models with successive addition of the relative amount of one more fatty acid, starting with myristic acid. We were especially interested in changes in the standardized beta coefficient (beta) when adding extra fatty acids to the list of independents. Results are presented as tables showing mean values ± SEM, or as scatter plots with the regression line included. SPSS 19.0 was used for the regression analyses and Sigma Plot 2001 for producing the figures. A significance level of 0.05 was accepted.

## Competing interests

Both authors declared that they have no competing interests.

## Authors’ contributions

ATH conceived and designed the study, analyzed and interpreted the data, and drafted the article. AH contributed substantially to the interpretation of data and revising it critically for important intellectual content. Both the authors approved the final version to be published.
